# Research Progress on Metal Ion Recovery Based on Membrane Technology and Adsorption Synergy

**DOI:** 10.3390/ma17143562

**Published:** 2024-07-18

**Authors:** Yiqing Feng, Rui Wang

**Affiliations:** School of Environmental Science and Engineering, Shandong University, Qingdao 266237, China

**Keywords:** membrane technology, adsorption, metal recovery, nanofibrous membranes, MXene, water treatment

## Abstract

The development of modern industry will generate more and more waste containing metal ions. It is necessary to take appropriate measures to recover these ions, whether from the perspective of environmental protection or improving economic benefits. So far, scientists have studied many methods for recovering metal ions. Among these methods, adsorption and membrane separation have received widespread attention due to their own characteristics. Combining adsorption and membrane separation methods can better leverage their respective advantages to improve the ability of recovering metal ions. This review, therefore, focuses on the synergistic recovery of metal ions by adsorption and membrane separation methods. This article first briefly explains the theoretical principles of membrane separation and adsorption synergy, and then focuses on several technologies that have received attention in different chapters. In these chapters, membrane technology is briefly introduced, followed by the situation and progress of synergistic application with adsorption technology. Then, the article compares and elaborates on the advantages and disadvantages of the above technologies, and finally summarizes and looks forward to these technologies being used to solve the difficulties and challenges in industrial application.

## 1. Introduction

Metal recovery is significant due to its environmental benefits and its contribution to competitive economic systems [1]. Heavy industries such as mechanical manufacturing, metallurgy, and chemical engineering typically generate a large amount of dust and wastewater containing heavy metals. These substances, including cadmium (Cd), chromium (Cr), lead (Pb), mercury (Hg), etc., enter the ecosystem through the atmosphere or water cycle, even posing a threat to animals, plants and humans through the food chain [2]. On the other hand, China requires a large amount of mineral resources every year to meet industrial demand. For example, China imported 4.415 million tons of lithium ore in 2023, according to news reports. Coupled with enormous amounts of scrap metals awaiting recycling, increasing mineral imports does not achieve carbon neutrality goals. Therefore, how to deal with heavy metal pollution and improve metal utilization is currently a hot topic in the environmental field.

Various methods are employed for the recovery of metal ions, categorized into physical methods like adsorption [3], membrane separation [4] and ion exchange [5]; chemical methods such as oxidation-reduction [6], electrodialysis [7] and chemical precipitation [8]; and biological methods including plant absorption, microbial treatment [9] and algae treatment [10].

**Figure 1:**
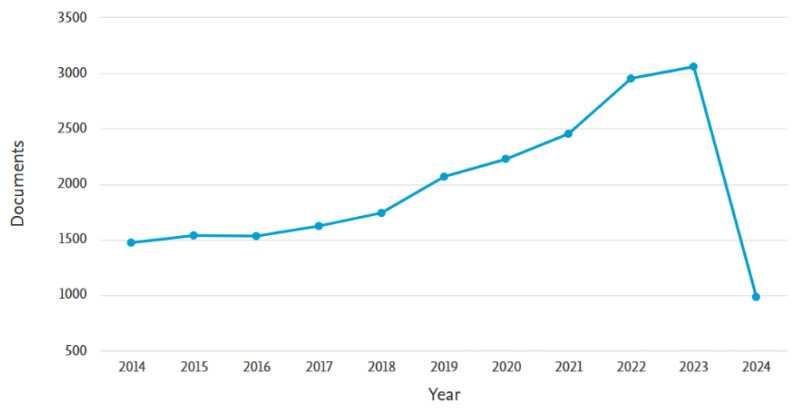
Number of documents found using the keywords “membrane” and “adsorption”. Sources: Scopus from Elsevier (until 20 March 2024).

Among the above methods, adsorption is widely used due to its high efficiency, simple operation, low cost, good stability, and diverse selection of adsorbents [4,11,12,13]. The key is to choose appropriate adsorption materials. The adsorption materials used for metal ion recovery mainly include activated carbon [14], zeolite [15] and graphene [16]. Also, membrane materials have been extensively employed due to their diverse types, high performance and selectivity, simple modification, mild operation and low chemical consumption [11,17,18,19]. However, the two methods have their own disadvantages, so some scientists combine membrane technology with adsorption technology and synergistically apply it to metal ion recovery. This not only overcomes the limitations of a single technology, but also leverages their respective advantages to achieve synergistic effects of technology, and improves the efficiency and economy of metal ion recovery. An overall analysis of the literature in the Scopus database, especially for the past three years, reveals that the synergy of the two technologies has generated much interest in metal recovery in the past decade. Statistics are as shown in Figure 1.

This review first analyzes the mechanism of membrane technology and adsorption synergistic recovery of metal ions from a theoretical perspective. Then this paper discusses the concepts, classifications, and application progress of several membrane technologies separately. Next, the article compares the membrane technologies discussed and analyze their advantages and limitations. Finally, a summary of this type of membrane technology is provided, and its potential applications in the industrial field are discussed. 

## 2. Theory of Membrane and Adsorption Synergy Technology

Membrane technologies are widely used in metal recovery because of its easy operation and high efficiency. In theory, a membrane can be seen as a barrier that blocks the passage of certain metal components (i.e., retentate) while allowing other components (i.e., permeate) to pass through. Therefore, the removal mechanism of membrane separation mainly involves size exclusion, as well as Donnan exclusion (charge–charge repulsion) effect and adsorption capacity for specific pollutants [4].

In addition to size exclusion, adsorption phenomena also can play a part in metal-polluted water treatments and recovery by membranes. Adsorptive membranes are a modification of existing membranes (e.g., ultrafiltration (UF) membranes) by adding additives to improve membrane properties such as hydrophilicity, porosity, mechanical strength and chemical stability [20,21]. For instance, the UF membrane can be added to some functional groups or compounds that can coordinate with metals (-NH_2_, -COOH, MOF, etc.) to achieve a membrane’s adsorption abilities [22,23,24]. Therefore, the mechanism of membrane adsorption is like traditional adsorption, where the adsorbent transfers substances from the liquid phase to the solid phase by physical or chemical processes [22]. Many studies have also evaluated membrane adsorption behavior and tried to find the adsorption process and mechanism of membranes through isothermal and kinetic analysis methods [22,25,26,27].

**Figure 2:**
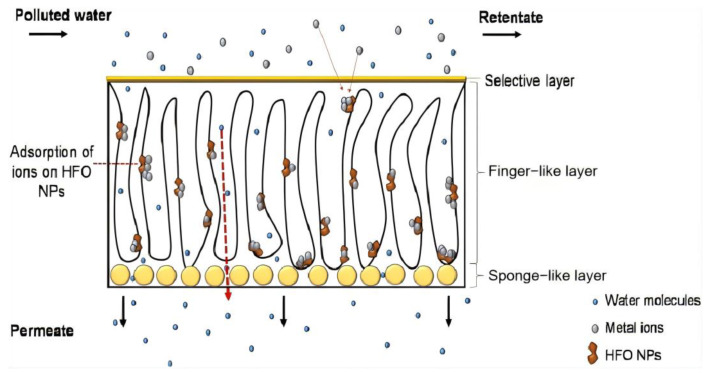
Illustrated mechanism of heavy metals adsorption on adsorbents in UF MMMs. Reprinted from [4].

Abdullah et al. [20] reported that although the membrane pore size is smaller after adding adsorptive nanomaterials, the adsorption process is the main removal mechanism, instead of size exclusion. As metal ions are still smaller than the pore size of modified membranes, ions can permeate membranes. This also means that adsorption performance is important for ion removal. The adsorption of metal ions onto mixed matrix membranes (MMMs) may involve four possible steps in the study: (1) bulk diffusion of ions into membrane pores and finger-like layer; (2) inner sphere surface complexation on the outer sites of adsorbents; (3) transfer of ions across the adsorbent interface; and (4) equilibrium stage. Therefore, the rate-controlling step for the sorption of metal ions is chemisorption on the external active sites of adsorbents, while the rate-limiting step is the transfer of ions across the adsorbent interface [20], as shown in Figure 2.

## 3. Metal Recovery by Different Membrane and Adsorption Synergy Processes

### 3.1. Ultrafiltration Membranes

Ultrafiltration (UF) membranes work at a low trans-membrane pressure to remove dissolved and colloidal materials. Because the pore sizes of UF membranes are larger than metal ions dissolved in aqueous solutions in the form of ions or complexes, these ions can pass through UF membranes easily [8]. As mentioned above, certain functional groups or compounds could be added to UF membranes to increase their adsorption efficiency. UF can be divided into two sub-UF processes: polymer-enhanced UF (PEUF) and micellar-enhanced UF (MEUF). 

**Figure 3:**
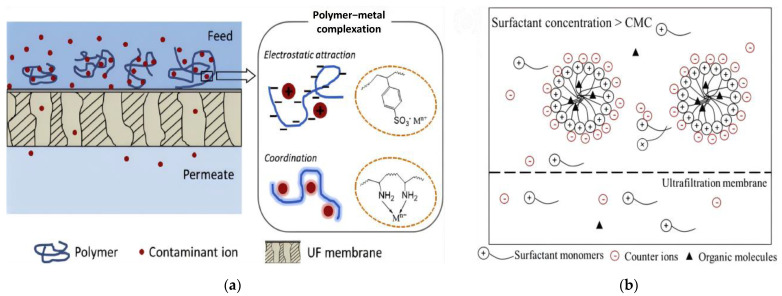
(**a**) Illustration of PEUF. Reprinted from [30]. (**b**) Illustration of MEUF. Reprinted from [32].

PEUF was initially formed by the complexation of polysaccharides extracted from biomass with metal ions [28]. The mechanism of PEUF is to form a complex between polymer ligands and metal ions, making the metal ion larger than the membrane pores and achieving a separation process [29]; as illustrated in Figure 3a [30]. In fact, the selection of the polymer ligands in PEUF is very important to improve the combination between metal ions and polymers. Polymer reagents need to meet the following requirements: high affinity towards target metal ions; high molecular mass; high water wettability; chemical and mechanical stability; and low toxicity and costs [31]. If the above standards cannot be met, metal ions will penetrate the membrane, leading to a decrease in recovery rate. 

Similar to PEUF, the MEUF process is also used to prevent metal ions from passing through UF membranes, but this process is achieved by forming micelles between metal ions and surfactants; as depicted in Figure 3b [32]. That is to say, cationic or anionic surfactants should be added according to the situation of the water body when designing MEUF. When the concentration of surfactant exceeds the critical micelle concentration (CMC), surfactant monomers aggregate with metal ions to form amphiphilic transparent micelles, and the hydrodynamic diameter will be larger than the pores of the UF membrane. In addition, some parameters should be considered when performing MEUF; such as pressure, pH, concentration and dosage of active agents, and the metals to be removed [4]. 

Through the above introduction and analysis, it should be noted that UF membranes themselves have a certain ability to trap metal ions through polymerization or ion interactions, but this ability is not strong. Membrane modification can be achieved by inoculating adsorbent materials on the surface of the membrane, which allows the UF membrane to play its own role while also improving rejection efficiency through surface adsorption. This is the fundamental essence of the so-called synergistic effect.

**Figure 4:**
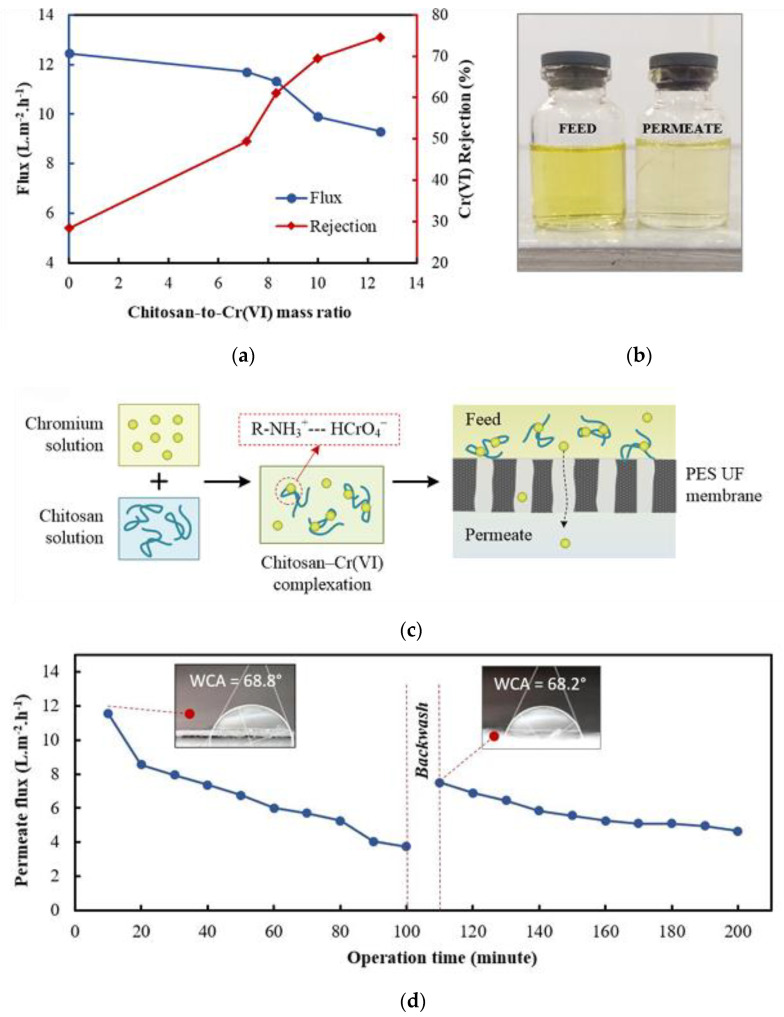
(**a**) Effect of the chitosan/Cr(VI) ratio on PEUF Performance. (**b**) Feed and permeate solutions obtained after PEUF using chitosan/Cr(VI) ratio of 12.5. (**c**) Schematic illustration of chitosan-Cr(VI) complexation and the enhanced removal in the UF membrane process. (**d**) Permeate flux before and after backwashing in PEUF for 200 min at 1 bar pressure. The insets show the contact angle of water droplet on membrane surface. Reprinted from [36].

Some recent studies conducted on UF for the removal of metal ions are presented in Table 1. From the table, it can be seen that various polymers or surfactants can functionalize UF membranes and improve their ability to retain metals. For instance, chitosan—the most important derivative of the natural amino polysaccharide chitin—is receiving increasing attention due to its high binding ability, wide availability and unique functions [33,34,35]. Indiasih et al. combined chitosan as a polymer with PES membrane and studied the effect of the ratio of chitosan to hexavalent chromium Cr(VI) on its rejection rate and permeation flux [36]. The results showed that when chitosan with a mass ratio of 12.5 of chitosan/Cr(VI) was added, the rejection rate of Cr(VI) rapidly increased from 28% to 75%, while the permeation flux only decreased by less than a quarter. The authors believe that the mechanism of increased rejection is that the amino groups of the chitosan used can achieve complete protonation at pH 5. The protonated amine group combines with Cr(VI) to form a chitosan-Cr(VI) complex, as shown in Figure 4c, which achieves adsorption of Cr(VI) in the form of the complex. The number of protonated amine groups also increases with the increase of chitosan mass, thereby enhancing the formation of complex macromolecules. Combined with the separation effect of the membrane, the interception of metal ions is completed. In addition, the color change between the feed solution and the permeate is shown in Figure 4b. This experiment demonstrates the excellent ability of chitosan. Some experts have also carried out functionalization modifications on the basis of chitosan to enhance the ability of UF membranes to recover metals. Nikhil Chandra et al. studied the functionalization of chitosan/poly (styrene sulfonate) (CHS/PSS) polyelectrolyte multilayer membranes (PEM) with tannic acid (TA) and campesterol (CHT) under UF conditions, and investigated the ability of these membranes to recover lithium [37]. The results showed that TA and CHT were immobilized on PEM membranes and achieved a high recovery rate of over 94% and excellent permeation flux. Through a series of experiments, it was also found that the bare membrane itself repels cations due to the surface charge of the membrane. The addition of TA or CHT causes the surface of the membrane to form a porous structure, increasing the surface area of the membrane. At the same time, it also allows metal ions to interact with carboxyl groups in TA or CHT to form complexes. These processes allow metal ions to be trapped inside the membrane. In addition, high cycling performance and lifespan also demonstrate the potential application of functionalized PEM.

**Figure 5:**
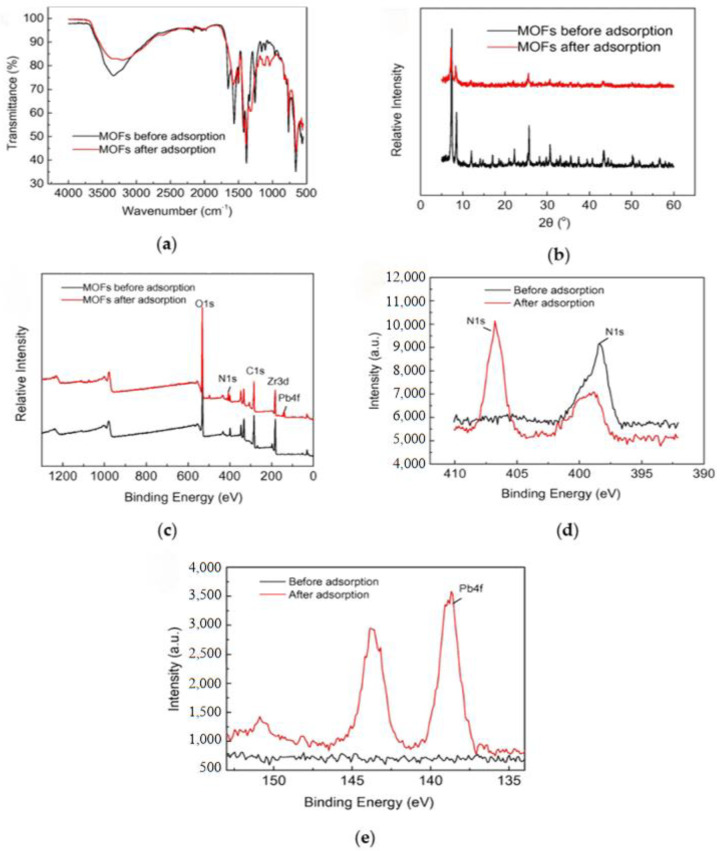
Adsorption mechanisms of Pb(II) onto the MOFs (**a**) FT-IR; (**b**) XRD patterns; (**c**) survey XPS; (**d**) N (1s); and (**e**) Pb (4f) spectra before and after adsorption. Reprinted from [42].

Apart from chitosan, metal organic frameworks (MOFs) are widely used to modify UF membranes to strengthen the ability of metal recovery. Yin et al. functionalized Zr-MOFs with amino groups and combined them with ceramic membrane ultrafiltration (CUF) to remove Pb(II) from wastewater [42]. Some results are shown in Figure 5. The membrane separation process of the aqueous solutions was achieved at a high trans-membrane pressure of 0.23 MPa and a low cross-flow velocity of 3.0 m·s^−1^. MOFs nanomolecules on the CUF adsorbed Pb(II) during this process, achieving the synergistic application of the two technologies. It was found that the removal rate of lead by the composite membrane exceeded 60%, and the adsorption capacity also reached 1795.3 mg/g. This successfully verified the adsorption mechanism of the coordination interaction between the amide group (-NH_2_) and Pb(II). Additionally, after cleaning the membrane, its recovery efficiency approached 100%. Senol-Arslan et al. used a solvothermal method to prepare Ni-Zn-MOF powder and prepared Ni-Zn-MOF-embedding membranes with different concentrations [43]. Similar to reference [42], the Ni-Zn-MOF membrane not only exhibits ion interactions on the membrane surface, but also possesses the adsorption capacity of MOF, enabling higher rejection rates during the separation process. The influence of temperature, pH and other parameters on the adsorption capacity of Pb(II) was studied through a series of characterizations. It was found that the MOF-modified PSF membrane had a better Pb(II) rejection rate than the pure membrane, and reached 98% under optimal conditions. 

Adsorptive ultrafiltration mixed matrix membranes (MMMs) are a new strategy to remove metal cations, which achieve ultrafiltration and adsorption functions in one unit. Adsorptive ultrafiltration MMMs can be divided into four categories, according to the types of adsorbents: inorganic filler, organic filler, biomaterial and mixed filler membrane [47]. Kumar et al. prepared a novel polyethylene tetrazole co-polyacrylonitrile (PVT-co-PAN) membrane using a nonsolvent induced phase separation (NIPS) method [26]. After adding PVT fragments, the membrane became more negatively charged and hydrophilic, and PVT fragments are the main binding sites for adsorbing Cu(II) ions, with an adsorption capacity of 44.3 mg/g; higher than other membranes reported in the literature. Usman Farid et al. [48] performed Ar/O_2_ plasma treatment on multi-walled carbon nanotubes (MWCNTs) [49]. The experimental results showed that the composite membrane with functionalized MWCNTs maintains an adsorption and removal efficiency of over 90% for Zn^2+^. In addition, selective adsorption of zinc can also be achieved in the presence of common coexisting ions such as Na^+^, Fe^2+^, Mn^2+^, Mg^2+^ and Ca^2+^. Importantly, the adsorption capacity of the CNT membrane for zinc can be easily regenerated under acidic conditions.

### 3.2. Nanofiltration Membranes

Nanofiltration (NF) is a technology between UF and RO and its pore size is in the range of 1–10 nm [50]. The advantages of NF membranes are their low energy demand, high efficiency for metal removal, easy operation and low pressure requirement [8]. NF membranes are generally made of aliphatic amine monomers, which carry positive or negative charges on the membrane. Due to the electrostatic interaction between metal ions and the membrane, coupled with the loose structure and small pore size of NF membranes, the separation and recovery of metal ions can be well achieved [4,51]. In addition to commercial NF membranes, existing substances or processes can be used to improve NF membranes, achieving the goal of enhancing their ability to adsorb metal ions. Some recent studies about NF membranes are shown in Table 2.

**Figure 6:**
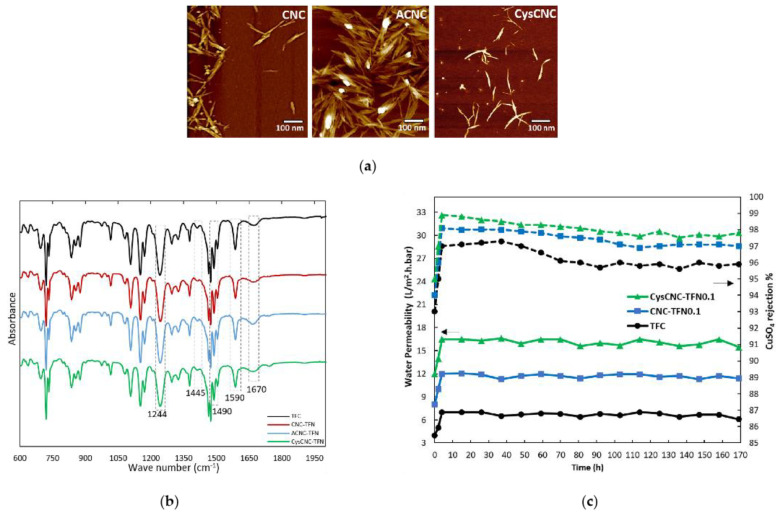
(**a**) AFM images of freeze-dried CNCs, ACNCs and CysCNCs. (**b**) ATR-FTIR spectra of TFC and TFN membranes in 0.1 wt% CNCs, ACNCs and CysCNCs. (**c**) Long-term performance test of TFC, CNC-TFN0.1 and CysCNC-TFN0.1 membranes within 200 ppm CuSO_4_ solution as a feed. Left arrow: water permeability of the three bottom lines; Right arrow: rejection rate of CuSO_4_ on the top three broken lines. Reprinted from [49].

Xu et al. [63] developed a positively charged nanofiltration membrane with a dense polyamide (PA) layer and surfactant functional layer (SFL); namely, the CTAB/PIP/TMC TFC membrane. Hexadecyltrimethylammonium bromide (CTAB) endowed it with a superior positively charged surface, resulting in a separation efficiency of over 98% for Zn^2+^ and Ni^2+^, over 96–98% for Cu^2+^ and over 85% for Pb^2+^. It also found that CTAB molecules form multilayers through electrostatic adsorption and hydrophobic interactions, changing the surface properties such as pore size, surface charge, and chemical composition of the membranes. Most metal ions are blocked due to charge repulsion. Even if a small number of metal ions reach the PA layer, the carboxyl groups on the surface of the PA layer will combine with the metal ions to complete the adsorption process. Abedi et al. synthesized l-cysteine functionalized cellulose nanocrystals (CysCNCs) using the iodine catalysis method, and dispersed the CNCs into trimeric chloride in n-hexane for heavy metal removal experiments [49]. After evaluating the membrane performance of thin film composite materials (TFC); and CNC, ACNC and CysCNC TFN membranes, it was found that the CysCNC-TFN0.2 membrane exhibited the best heavy metal removal performance, of over 85%. The analysis and results are shown in Figure 6. The reason for achieving such high removal efficiency is that NF membranes mainly rely on electrostatic repulsion and size repulsion to separate metals. The thiol (-SH) functional groups of CysCNCs introduce additional sites to enhance the repulsion of heavy metals through complexation or adsorption [64]. The coordination interaction between Cu-S and Pb-S (chelation effect) can lead to higher repulsion of Cu^2+^ and Pb^2+^ metal cations. They further speculate that, as part of the Lewis soft base family, sulfur-containing species have highly polarized donor centers that can strongly react with the orbitals of soft Lewis acids (such as heavy metal ions). Furthermore, the improvement of TFN membrane performance was mainly due to the functionality of nanoparticles and the uniform distribution of particles dispersed in the polyamide layer through solution.

Roy et al. used simple impregnation and cross-flow filtration methods to modify polyethylene imine (PEI) and EDTA, and prepared new amine functionalized composite membranes P-60S and P-60S-EDTA on a tubular ceramic substrate [65]. The experimental results showed that P-60S-EDTA has superior permeability and rejection efficiency, with rejection rates of over 90% for Cu(II), As(V) and Cr(VI). The membrane operated well in both ideal and actual environments. This is because, in the case of the P-60S-EDTA composite membrane, the reduction of pore size and the presence of surface functional groups on the membrane are conducive to the interception of spatial effects. Under low pressure and pH 3 conditions, the chelation of HCrO_4_^−^ and H_2_AsO_4_^−^ ions with protonated surface amino groups is the main mechanism for removing As(V) and Cr(VI). However, as the operating pressure increases, small anions will pass through the composite membrane, leading to a decrease in rejection rate and an increase in the number of discharged anions.

Arouche et al. used carbon nanotubes and boron nitride nanotubes as nanofiltration membranes, and tested the membrane’s rejection effect on heavy metal ions through electric field force testing [66]. The study showed that both nanotubes had the best filtration effect when the electric field strength was 10^−8^ a.u. The kinetic energy and in situ temperature required for both types of nanotube filtration are the minimum values in 10^−8^ a.u. Due to differences in hydrophobicity and thermal stability, the effect of boron nitride nanotubes (90–98%) was better than that of carbon’s (80–90%). Meanwhile, Arouche et al. also found that as the electric field (EF) strength increases, the increase in evanescent effect will affect the treatment effect. Based on this, they believe that nanofiltration will have better results at lower EF strengths. An additional inference is that the filtering effect may be better without adding any EF.

**Figure 7:**
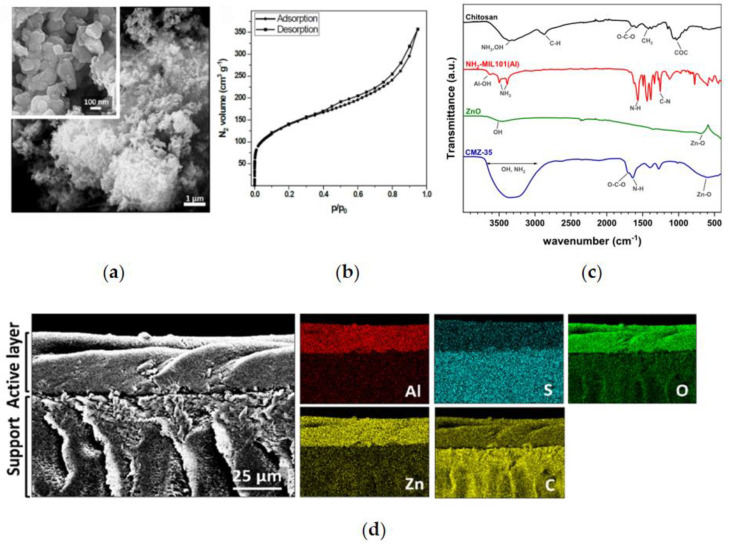
NH_2_-MIL-101(Al) powder: (**a**) low-magnification and high-magnification (in the insert) SEM micrographs, and (**b**) nitrogen adsorption/desorption isotherms (77 K). (**c**) FTIR analysis of chitosan, NH_2_-MIL101(Al), ZnO, and CMZ-35 nanocomposite. (**d**) EDX analysis of the CMZ-35 membrane. Reprinted from [59].

**Figure 8:**
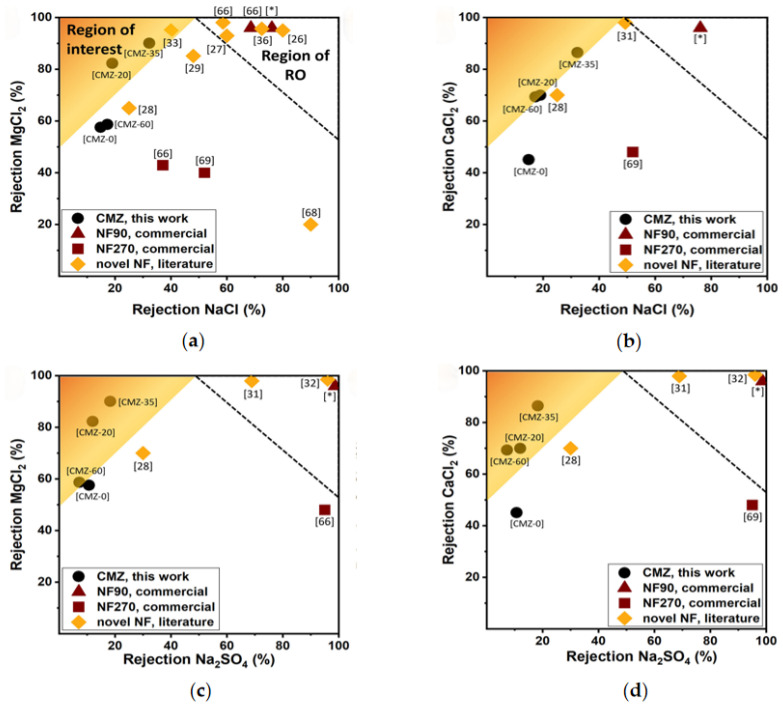
For article [59], the results are compared with Fang et al. (2013) [68], Feng et al. (2014) [69], Li et al. (2006) [70], Li et al. (2012) [71], Liu et al. (2015) [72], Wu et al. (2016) [73], Wu et al. (2014) [74], Xu et al. (2016) [75] and Zhang et al. (2017) [76]. And the salt couples: (**a**) MgCl_2_/NaCl, (**b**) CaCl_2_/NaCl, (**c**) MgCl_2_/Na_2_SO_4_, and (**d**) CaCl_2_/Na_2_SO_4_ at operating pressures about rejections for CMZ membranes in this work, commercial NF membranes, and positively charged NF membranes in the literature. Region of interest: high rejection values towards MgCl_2_ and CaCl_2_ with respect to NaCl and Na_2_SO_4_; Region of RO: typical rejection values for RO membranes. * Tested in their work.

Fonseka et al. [67] modified the chromium-based MOF with N-(phosphonylmethyl) iminodiacetic acid (PMIDA) and amine grafted mesoporous silica (SBA15) materials for selective recovery of rare earth elements (REE) and copper, respectively. Two adsorbents were used sequentially to selectively adsorb REE (91%) and Cu (90%) from the concentrated feed with pH regulation. The main driving force for adsorbing REE is the formation of coordination complexes with carboxylate and phosphine ions on the MOF; and the adsorption of Cu is due to the strong chelation bond between Cu and amine ligands. Morgante et al. [59] incorporated the active layer of NH_2_-MIL-101 (Al) and ZnO nanoparticles into the chitosan matrix, and combined it with the ultrafiltration matrix to prepare a nanofiltration membrane. The characterization analysis and results are shown in Figure 7 and Figure 8. The experiment found that the membrane containing 35 wt% ZnO had the highest rejection rates for MgCl_2_ (90.10%) and CaCl_2_ (86.49%) at a solution concentration of 1000 ppm, and the selectivity for these two substances was higher than that of commercial membranes (NF90 and NF270). 

### 3.3. Electrospun Nanofibrous Membranes

The electrospun nanofiber membrane (ENFM) is essentially a polymer-based membrane [77]. Polymer-based membranes can be prepared using various methods, including stretching, phase separation, electrospinning, evaporation, and etching [78]. Among these methods, electrospinning is a simple, reliable, universal, and sustainable technology. The significant characteristics of spinning nanofibers are high surface-to-volume ratio, high porous structure and excellent connectivity, sufficient flexibility, extensive material collection, and the ability to prepare different structures by adding different materials through electrospinning [79,80]. According to Uddin et al.’s article, there are six preparation methods for electrospinning [80]. For convenience, these membranes made from electrospun fibers by these methods are usually referred to as “electrospun nanofiber membranes (ENFMs)”. Since ENFMs exhibit excellent adsorption performance because of their high specific surface area and high porosity, ENFM is a good choice for removing heavy metals from aqueous solutions [77]. Adsorbents require active sites so that metals can be removed through ion interactions or chelation. At present, researchers use CS, cyclodextrin (CD), polyacrylic acid (PAA), polyethyleneimine (PEI), polyindole (PIN) and polyaniline as nanofiber membranes to remove some metal ions [81]. However, most polymers have poor spinnability and stability in aqueous media. Therefore, by combining different polymers with various nanoparticles to improve their performance and stability, electrospun nanofiber composite membranes have been developed [81].

**Figure 9:**
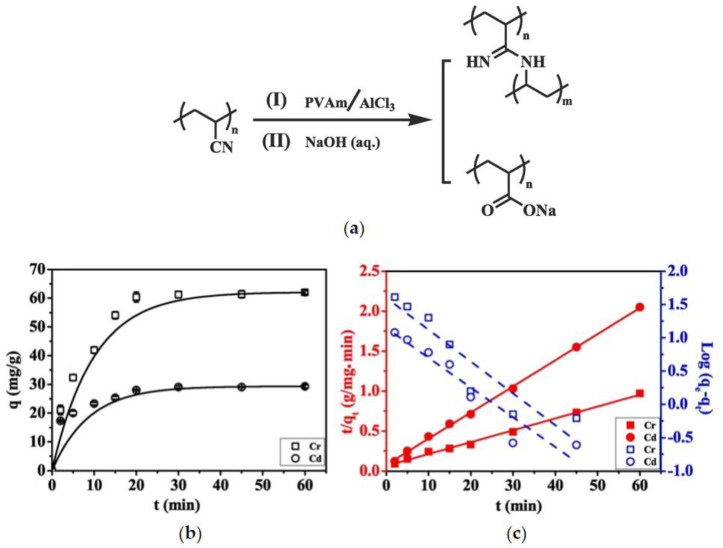
(**a**) Surface modification routes of PAN nanofibrous component. (**b**) Increase in the adsorption capacity (q) against Cr (VI) and Cd (II) ions with time using PVAm-g-PAN-PVA and hydrolyzed PAN-PVA composite membranes, respectively; and (**c**) corresponding pseudo-first order (logq_e_ − q_t_) and pseudo-second order (t/q_t_) kinetics plots of the adsorption processes. Reprinted from [82].

Liu et al. [82] prepared electrospun nanofiber composite PAN–PVA membranes and nanofiber PAN and PVA membranes, fabricated through a two-nozzle electrospinning process. Different modification methods were used to functionalize the PAN components of PAN–PVA nanofiber composite membranes: one was to use amino groups as adsorption sites to attract negatively charged heavy metal ions (such as Cr(VI)), as shown in reaction (I) in Figure 9a. The other method was to hydrolyze PAN with strong alkali to form sodium polyacrylate (as shown in reaction (II) in Figure 9a), and the resulting carboxylate ions coordinated with positively charged heavy metal ions, such as Cd(II). Using GA as a crosslinking agent to chemically crosslink PVA nanofiber components and modifying PAN, subsequently, the composite membrane was used for adsorption experiments of Cr(VI) and Cd(II); this achieved high results (66.5 mg/(g membrane) against Cr(VI) and 33.6 mg/(g membrane) against Cd(II)).

**Figure 10:**
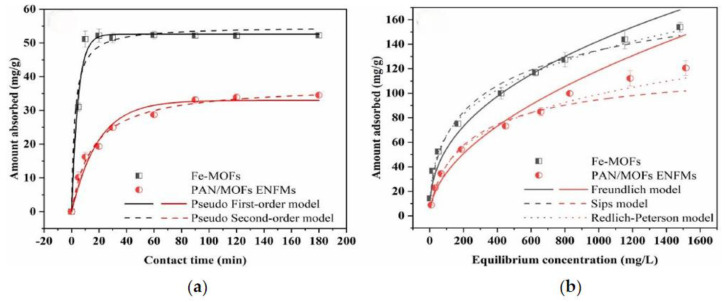
(**a**) Adsorption kinetics of Cr(VI) on Fe-MOFs particles and PAN/MOF ENFMs fitted by pseudo-first order model and pseudo-second order model; (**b**) Adsorption isotherms of Cr(VI) on Fe-MOFs particles and PAN/MOF ENFMs fitted by Freundlich model, Sips model and Redlich-Peterson model. Reprinted from [83].

Miao et al. [83] first synthesized iron-based MOFs (Fe-MOFs), and then co-electrospun polyacrylonitrile (PAN) to prepare composite electrospun nanofiber membranes (PAN/MOFs ENFMs). In the co-electrospinning process, ENFMs mainly act as carriers, and MOF particles are the main adsorbents. Fe-MOFs particles are fixed in situ in ENFMs, with MOFs particles partially on the fiber surface and partially encapsulated within the fiber, forming complex porous structures. The results showed that PAN/MOFs ENFMs had a maximum theoretical adsorption capacity of 127.70 mg/g for Cr(VI) at pH 4.0, and partially adsorbed Cr(VI) could be reduced to less toxic Cr(III). The above results are shown in Figure 10. It was also found that the adsorption of Cr(VI) is a spontaneous process dominated by physical adsorption. In Miao’s article, the maximum theoretical adsorption capacity is determined based on the saturation adsorption capacity of the theoretical isotherm, and its value is calculated using the Sips model. The formula is as follows [83]:

q_e_ = q_m_ × (a × C_e_)^1/n^/(1 + (a × C_e_)^1/n^)

a: Sips affinity coefficient; 1/n: Sips affinity coefficient.

Additionally, Mohammed et al. [84] developed a PAN/PEI nanofiber composite membrane (NCM) based on ZIF-8 nanoparticles, and then the adsorption of Cr(VI) in water by NCM was tested. The results showed that the addition of PEI endows the surface of NCM with superhydrophilicity, while the adsorption capacity of NCM is 403.5 mg/g at a ZIF-8 concentration of 5 wt%. Through FTIR and XRD spectra, it was found that the adsorption process of Cr(VI) mainly involves the formation of hydroxyl (-OH), amino (-NH_2_), amide (-CO-NH) and other structures between PEI and PAN. ZIF-8 first adsorbs Cr(VI), and then these functional groups convert Cr(VI) to Cr(III) through redox reactions. Subsequent analysis also indicated that PAN/PEI@ZIF-8NCM has high selectivity and stability for wastewater treatment.

In addition, Yin et al. [85] used a dual nozzle electrospinning method to prepare electrospun sulfonated polyether sulfone (SPES) nanofiber membranes, and tested the fiber membrane’s adsorption capacity for heavy metal ions by adjusting the fiber diameter. In the ATR-FTIR spectrum of SPES, two new peaks were generated, representing the generation of –SO_3_H, which is served as adsorption sites for positively charged substances (e.g., Pb(II)). It was found that the rejection rate of Pb(II) by ultrafine nanofiber SPES membrane was 96.2% (the initial concentration of Pb(II) is 1.0 ppm), and the sustainability of the membrane was demonstrated in cyclic experiments. Li et al. [86] used HPEI grafted graphene oxide (GO) and polyacrylonitrile (PAN) to synthesize PAN/GO-HPEI electrospun nanocomposite fiber membrane through electrospinning. Then, HPEI was grafted through chemical grafting reaction to obtain HPEI-g-PAN/GO-HPEI electrospun nanocomposite fiber membrane, which was used as an adsorbent to adsorb Au(III) in aqueous solution. Due to the presence of amino groups, HPEI-g-PAN/GO-HPEI exhibited efficient adsorption of Au(III), with a maximum adsorption capacity of 2893.33 mg/g at the center point. This experiment also found that adsorbed Au(III) can be reduced to Au(I) and irregular thin and granular Au elements attached to the adsorbent.

### 3.4. Ion-Imprinted Membranes

**Figure 11:**
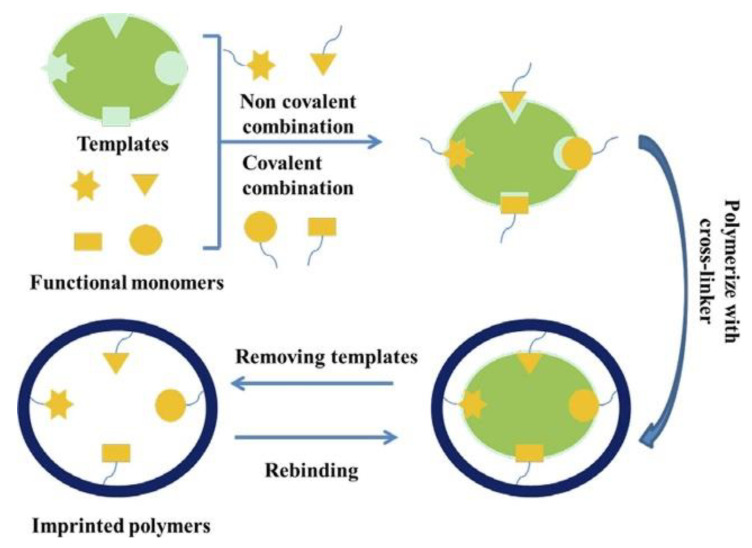
Preparation process and recognition mechanism of IIPs. Reprinted from [89].

Ion-imprinting technology (IIT) is derived from molecular imprinting technology, and the principles of these two technologies are similar; except that the template is replaced with ions. Although Dickey [87] proposed the concept of “specific adsorption” in 1949, it was not until 1976 that the application of ion imprinted polymers (IIPs) was first realized [88]. Since the emergence of IIPs, they have attracted attention due to their excellent chemical properties and specific selectivity. Template ions, functional monomers, crosslinking agents, initiators, and solvents are required to synthesize IIP; with the target generally being template ions. The preparation and identification process of IIP is shown in Figure 11. 

The ion imprinting membrane (IIM) is a combination of ion imprinting technology and membrane separation technology, which refers to a special functional membrane prepared by direct or modified synthesis between IIPs and membranes. IIM can achieve specific selectivity of IIT and high efficiency and stability of membrane separation, thereby achieving selective separation in complex solution systems. Compared with IIP, IIM can achieve the separation and concentration of metal ions without grinding, and demonstrates high recovery efficiency and low diffusion resistance [89]. In addition, Lu et al. [90] provided a more comprehensive introduction to the origin and development, mechanism, preparation, performance evaluation, application of IIM, etc.

**Figure 12:**
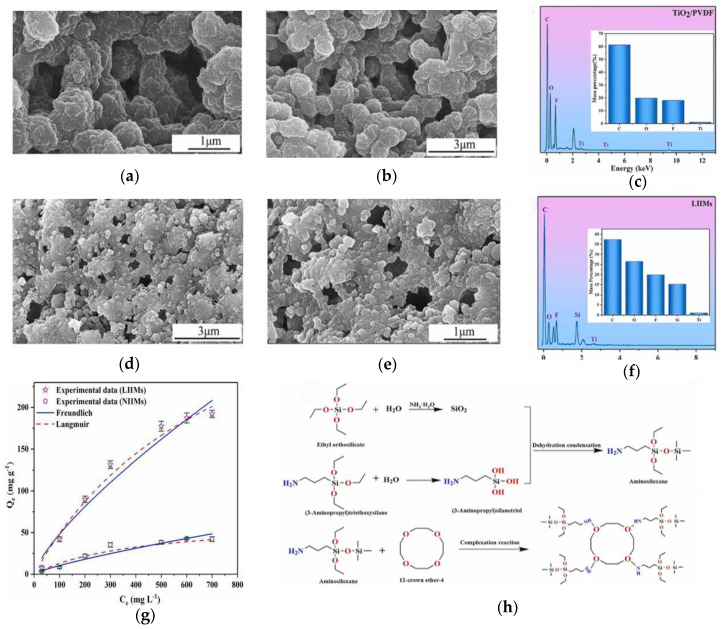
SEM images of (**a**,**b**) TiO_2_/PVDF, (**d**,**e**) LIIMs and EDS spectrum of (**c**) TiO_2_/PVDF and (**f**) LIIMs. (**g**) The nonlinear fitting curves of the Langmuir model and Freundlich model for the adsorption of LIIMs and NIIMs to Li^+^ (LiCl solution). (**h**) Schematic diagram of synthesis of LIIMs. Reprinted from [91].

Yang et al. [91] used 12-crown-4 ether (12C4) as a selective metal complexing agent for lithium ions and prepared lithium-ion imprinted membranes (LIIMs) through hydrolysis polymerization; the preparation process is shown in Figure 12h. Observing the SEM images, it can be seen that Figure 12d,e shows that LIIM has a rougher surface than Figure 12a,b. Many microspheres were loaded on the surface of lithium-ion imprinting membranes. It was also found that LIIM exhibited rapid adsorption kinetics and adsorption capacity (132.00 mg/g), and Langmuir isotherm adsorption results confirmed that the imprinting sites were uniform. Perm selectivity experiments and adsorption regeneration cycles demonstrated the excellent selectivity and stability of LIIM. He et al. [92] prepared a multi-layer interlayer (SiO_2_@SP-PDA) and even coat on the substrate surface (PVDF membrane). By modifying SiO_2_@SP-PDA with polydopamine (PDA), the aggregation of the ion imprinting layer was reduced while increasing the adsorption capacity to 231.77 mg/g. When analyzing the mechanism of selective adsorption, it was found that the peaks of C-O-C/C-O and O-C=O both shifted, and the intensity of the peaks significantly decreased, indicating that the O-containing functional groups of methacrylic acid (MAA) and 12C4 participated in the chelation of Li^+^. The changes in adsorption peaks observed in ATR-FTIR spectra before and after Li^+^ adsorption also confirm this viewpoint. Therefore, the adsorption of Li^+^ mainly depends on the chelation effect and appropriate spatial structure. Qu et al. [93] prepared a TiO_2_/PVDF composite LIIM using 12-crown-4 ether as a functional monomer. LIIMs showed high affinity for Li^+^ (799.82 mg/g, 308 K) in simulated leaching solution (SLS), and also showed high selectivity for Li^+^ in lithium and other ion systems. Through SEM, FT-IR and XPS analysis, it was found that the adsorption mechanism of LIIMs is the coordination between the active adsorption sites on the crown ether surface and Li^+^, and oxygen atoms provide lone electron pairs for Li^+^ to form coordination bonds.

Except for LIIMs, other metal ions can be used to make IIMs. For example, Jin et al. [94] developed an IIM to adsorb Ag^+^ ions in aqueous solutions. The dynamic adsorption results showed that the prepared IIM exhibited excellent performance (29.98 mg/g) when applied to feed solutions with 12 mg/L and pH = 6. The reason for achieving such results is, on the one hand, the FTIR spectrum showed that N-benzoyl-N’, N’—dibutylthiourea (BDBTU) had successfully adhered to the membrane; on the other hand, XPS and EDS spectra confirmed that the formation of chemical bonds between Ag^+^ and the -C(=O)-NH-C(=S)- group of BDBTU is believed to occur through electron transfer from the C=O and C=S groups of IIM to Ag^+^. This reaction enhances the adsorption capacity of Ag^+^ in the solution. Zhang et al. [95] developed an IIM by self-assembling pyridine monomers in restricted reduced graphene oxide (r-GO) nanochannels for Nd extraction. The membrane exhibited excellent Nd(III) adsorption capacity of 20.6 mg/g in a solution containing seven rare earth elements (REE). The ion radius, average bond length between Nd(III) and pyridine nitrogen, and the coordination of Nd(III) leading to an increase in nitrogen binding energy are the main factors for Nd(III) targeted binding to bipyridine nanocages. Zhang et al. [96] used chitosan to prepare two types of Nd-IIMs. The kinetic experiments on Nd(III) showed that both composite membranes reached 80% of their maximum adsorption capacity within 200 min. Even after 10 cycles of adsorption and desorption, the imprinted composite membrane still maintains a removal rate of about 80%. Mokhtar et al. [97] prepared an ion imprinted PVDF/diVB-TETA-Cu membrane, and a series of results indicate that the prepared membrane is suitable for the selective separation process of Cu^2+^ ions. In the selective experiment, it is believed that the selective adsorption of copper mainly involves the following steps: (1) the concentration difference between the feed side and the receiving side, which causes Cu^2+^ and Ni^2+^ to move from the solution to the membrane interface; (2) the chelation reaction between the active site and Cu^2+^, which is due to the higher affinity of TETA for Cu(II) and the higher imprinting effect of the membrane on Cu(II); (3) desorption into receiving compartment. Wang et al. [98] has prepared an ion imprinting multilayer membrane for selective separation of copper ions, which alternately assembles polyacrylic acid and chitosan to form a multi-layer structure. When the assembly layers are 3.5 layers of polyacrylic acid and 4 layers of chitosan, the adsorption capacity of Cu(II) is the highest (48.0 mg/g).

### 3.5. MXenes

**Figure 13:**
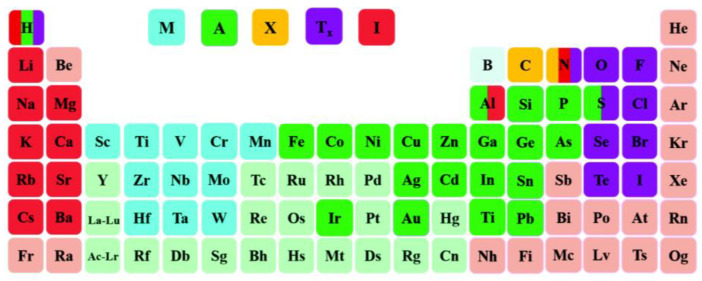
Intention of periodic table of elements related to MXene. Here, M, A, and X originate from the MAX phase, and Tx and I represent the surface termination groups and intercalants during the MAX-to-MXenes synthesis process (etching, intercalation or functionalization steps). Reprinted from [100].

**Figure 14:**
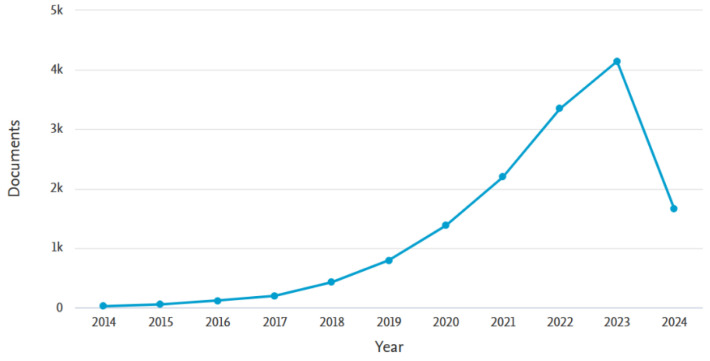
Number of documents on keywords “MXene” and “adsorption”. Sources: Scopus from Elsevier (until 20 March 2024).

MXenes refer to transition metal carbides and nitrides, which have become research hotspots in many fields as an emerging two-dimensional material [99]. The general formula of MXenes is M_n+1_X_n_T_x_ (*n* = 1, 2, or 3), where M represents transition metals (such as Mo, Cr, Nb, V, Zr, and Sc), X stands for nitrogen or carbon, and T refers to surface halogen or sulfur group element capped groups (such as -F, -OH, -O) [100]. MXene is mainly generated by removing the MAX phase of interlayer third or fourth group elements. MAX phase also refers to layered transition metal carbides and nitrides, which form M_n+1_AX_n_ (*n* = 1, 2, 3, and 4) with post transition metals of the III–VI group (such as Ni, Fe, Zn, and Cu) and A (such as Si, Al) [101]. Due to the difference in n, the allocation form of MAX phase is also different [102]. So far, many MXenes have been theoretically predicted, laying a solid foundation for extensive research on MXenes, as shown in Figure 13 and Figure 14. 

**Figure 15:**
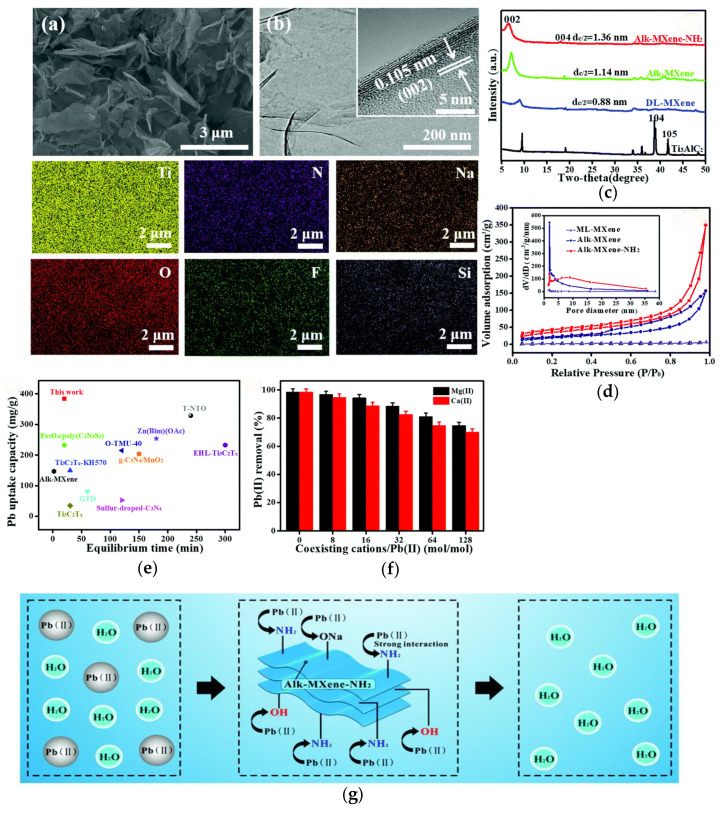
(**a**) SEM images of alk-MXene-NH_2_ and the corresponding element mapping of the alk-MXene-NH_2_ sample. (**b**) TEM and HRTEM images of alk-MXene-NH_2_. (**c**) The XRD patterns of the Ti_3_AlC_2,_ DL-MXene, alk-MXene and alk-MXene-NH_2_ sample. (**d**) N_2_ adsorption-desorption isotherm of ML-MXene powders, alk-MXene and alk-MXene-NH_2_ (insets are the corresponding BJH pore size distribution). (**e**) Effect of competitive ions on uptake of Pb(II) onto alk-MXene-NH_2_ (**f**). (**g**) The possible adsorption mechanism for alkMXene-NH_2_ to Pb(II). Reprinted from [104].

Currently, many studies have reported examples of MXene adsorbing metal ions (e.g., Pb, Cu, Cr, U). Powdered activated carbon (PAC) served as the control material for MXene (Ti_3_C_2_T_x_), and a series of methods were used to focus on the adsorption of Pb(II) by MXene. Jun et al. [103] found that the main adsorption mechanism of Pb(II) is electrostatic attraction in the experimental results of solutions with different pH levels, ion strengths, and humic acid concentrations. Furthermore, FT-IR and XPS revealed that the adsorption mechanism of Pb(II) is ion exchange and the formation of inner spherical complexes. In addition, the value of MXene in removing heavy metals was discovered through adsorption/desorption cycles. Zhang et al. [104] prepared Ti_3_C_2_T_x_ MXene nanosheets with amino groups through alkaline grafting modification based on the popular silane coupling agent (APTES). The prepared alk-MXene-NH_2_ increased the content of grafted organic functional groups, significantly improved interlayer spacing and chemical state, and exhibited strong adsorption capacity (384.63 mg/g). Biobased materials can also be used to improve the adsorption capacity of MXene due to their low toxicity and high biocompatibility. As a case example, enzymatic hydrolysis of lignin (EHL), lignosulfonate, and chitosan modified Ti_2_CT_x_ nanosheets were selected, and the differences in the removal of Pb (II) ions by various surfactants were analyzed [105]. The results showed that EHL functionalized Ti_2_CT_x_ exhibited excellent capacity for Pb(II) (232.9 mg/g) and prevented the re stacking of Ti_2_CT_x_ nanosheets. Therefore, enhancing the performance of activation sites is an effective strategy for enhancing the removal of Pb ions on MXenes. These above results are shown in Figure 15. 

**Figure 16:**
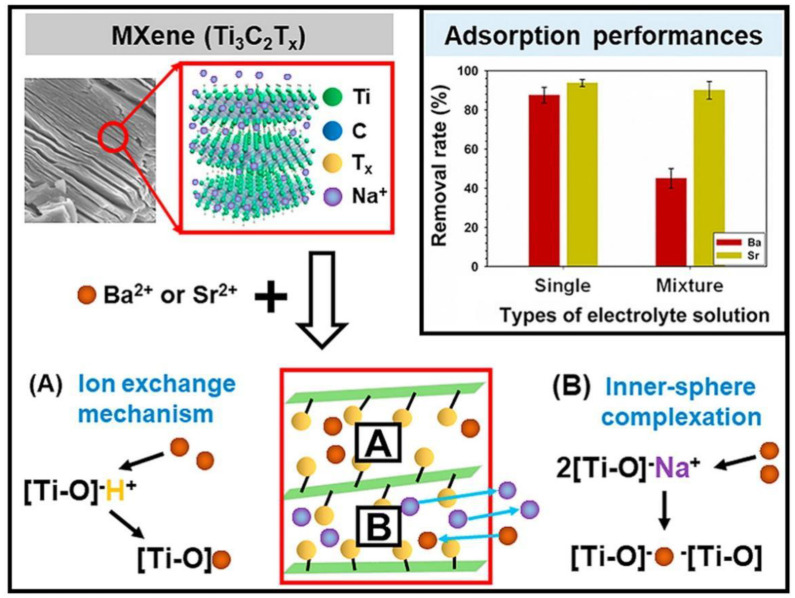
Adsorption mechanisms of Ba^2+^/Sr^2+^ by MXene and comparison of removal rate between Ba^2+^ and Sr^2+^ using single or mixture electrolyte solution: (**A**): ion exchange; (**B**): inner-sphere complexation (conditions: MXene dose = 100 g·L^−1;^ Ba^2+^ and Sr^2+^ concentration of each single or mixture electrolyte solution = 2 g·L^−1;^ temperature = 293 K; pH = 7; contact time = 1 h). Reprinted from [114].

MXene can also be used to adsorb other heavy metals and radioactive elements. Shahzad et al. [106] synthesized 2D Ti_3_CNT_x_ and Ti_3_C_2_T_x_ MXene nanosheets by peeling off Ti_3_AlCN and Ti_3_AlC_2_ MAX phases, respectively. It was found that during the adsorption of mercury ions, the interaction between bimetallic and hydroxyl groups with Hg^2+^ was achieved, and stronger adsorption ability was achieved (Ti_3_C_2_T_x_: 5473.13 mg/g; Ti_3_CNT_x_: 4606.04 mg/g). Apart from Hg^2+^ removal, Dong et al. used a new type of Mxene/alginate composite material, which has excellent removal ability for Cu^2+^ (87.6 mg/g) due to chemical coordination and ion exchange effects [107]. Elumalai et al. used amino acids to layer Ti_3_C_2_T_x_ thin films to study the removal of Cu^2+^ [108]. With the assistance of TiO_2_, histidine functionalized rutile TiO_2_@d-Ti_3_C_2_T_x_ The hybrid material achieves maximum adsorption capacity (95 mg/g) within 5 min. In addition, Kong et al. constructed amino functionalized MXenes (NH_2_-Ti_3_C_2_T_x_) using in situ polymerization of (3-aminopropyl) triethoxysilane (APTES) on the surface of Ti_3_C_2_T_x_ [109]. Through DFT calculation, the synergistic effect between Ti and N significantly improves the binding energy of MXene to Cr(VI) and the electron density on the surface of MXene. As for radioactive elements removal, a simple example is that Liu and co-workers reported the PEI-Ti_3_C_2_ exhibited huge uranyl loading capability, high removal efficiency and remarkable sequestration selectivity [110]. Shahzad et al. reported that Ti_3_C_2_T_x_ mixed Prussian blue aerogel (PBMX) spheres can effectively remove Cs^+^ (315.91 mg/g) from wastewater [111]. In addition, there are reports on the removal of Sr^2+^ and Ba^2+^ by MXene [112,113,114,115], as shown in Figure 16. 

## 4. Evaluation of Synergy Process

Table 3 compares the advantages and disadvantages of various membranes described in the previous chapters. In experimental design or industrial applications, it is necessary to find suitable membrane treatment methods based on the material composition and characteristics of the water to be treated. At the same time, it is also necessary to address the limitations of various membrane technologies in application to expand the scope of membrane applications.

Composite membranes should be concerned in terms of reusability. MMMs, IIMs, MOFs membranes and ENFMs all have high selectivity and adsorption capacity, but their cyclic regeneration performance is not good. Strong acids or alkalis are usually used to wash the membranes to improve their regeneration performance, but this will accelerate the aging of the membranes and reduce their service life. The nanomaterials in the composite membrane may also be leached, which can lead to a decrease in membrane treatment efficiency. On the other hand, after being discharged into the natural environment, nanoparticles may adsorb harmful substances in the environment, and may also promote the reaction among harmful substances to generate secondary pollutants. Many articles focus on the adsorption and regeneration capabilities of these composite membranes, with little attention paid to such environmental issues. For sustainability, there have been some attempts to use organic acids [119] and microorganisms [120] as leaching agents, but it will take some time for these new leaching agents to completely replace inorganic acids/bases. Also, the low dispersion rate of dissolved nanomaterials can cause the aggregation of nanoparticles, ultimately leading to the instability of membrane performance. 

For PEUF and MEUF, it is necessary to study the critical point of metal complexes or micelles. If the process is not improved, it may result in a very low removal rate of metal ions. In addition, waste of ligands/micelles and the production of harmful products may also occur. For ENFMs, it is important to study in detail its adsorption mechanism, the interaction between various substances in the composite membrane, the adsorption of multi-component solutions, and real sewage experiments [77]. For example, Nauman et al. [79] elaborated on the effects of processing parameters, solution characteristics, environmental factors and collector configuration on the mechanical strength, diameter, compatibility with adsorbents and other factors of electrospun fibers; and compiled many fiber post-treatment methods (such as crosslinking, hot pressing, hot stretching and solvent welding). For IIMs, ion recognition ability needs to be given special attention, mainly because the diameter of Mg^2+^ is similar to that of Li^+^, which creates competition when adsorbing Li^+^. In order to enhance selectivity, modified crown ethers [121], calixarenes [122], and blended chitosan [123] are generally used as chelating agents, and there have also been studies using “crown ethers + ionic liquids” [124,125]. MOF-based membranes often suffer from insufficient stability due to the different properties of the MOF layer and support layer, which limits their binding force and the continuous growth of the MOF layer. There are some methods to solve this problem, such as using interface growth-based methods to prepare Zn-MOF membranes, and developing sacrificial strategies for preparing MOF membranes containing water unstable MOFs (MOF-5, HKUST-1, and ZIF-67) [126]. Its biocompatibility and toxicity need to be studied for industrial application for MXenes. The toxicity of MXene based materials depends on their physical and chemical properties, such as size, dosage and surface coating; and surface chemistry is also an important factor affecting toxicity. Reducing toxicity can be achieved through size reduction [127], fluorine-free synthesis, and coupling with other materials [128,129]. 

It is worth mentioning that, although membranes are widely used in the field of metal ion separation and recovery, there are still some common challenges that affect the industrial application of membranes. For example, fouling, swelling, reusability and sustainability remain important factors determining the commercialization ability of any membrane. Therefore, if researchers can improve the membrane process and overcome the limitations of various membranes, membrane technology will become the main way of metal ion collection.

## 5. Conclusions and Prospects

Over the past several years, many methods have been developed to recover metal ions from aqueous solutions. Conventional and novel methods are applied with their own advantages and limitations. Membrane technology with adsorption ability exhibits huge potential amongst these methods, as it combines the advantages of membrane separation and adsorption. These include the UF membranes (PEUF, MEUF, MMMs), NF membranes, ENFMs, MOFs membranes, IIMs and MXenes. Numerous researches have reported positive results for metal recovery by membranes. For PEUF and MEUF, selecting appropriate complexing/micelle agents is vital to achieving high removal efficacy of metal ions and ensuring feasibility without generating excessive releases of secondary pollutants. Adsorptive UF MMM is suitable for treating low concentrations of metals by enabling the complete filtration-adsorption removal of metal ions to occur at low membrane pressure. ENFM is an environmentally friendly membrane and shows abilities for metal removal, but it is still necessary to address the issues related to the adsorption mechanism. IIM needs to further improve its ion recognition ability and usability, and develop more environmentally friendly regeneration methods. Similarly, MOF-based/modified membranes and MXene also need to address their regeneration ability and stability, as well as potential secondary environmental issues caused by the detachment and agglomeration of nanoparticles. These promising technologies, if their limitations and performance are improved while reducing usage costs, can be rapidly applied in the industrial field and eventually become a universal and sustainable method for metal ion recovery in the future.

## Figures and Tables

**Table 1:** Removal of heavy metals by UF membranes.

Membrane Systems	Metal(s) Studied	Flux (L/m^2^ h)	Concentration of Metal (mg/L)	Rejection (%)	Ref.
TA/chitosan/PSS/PEM	Li^+^	52.08	7	93.42	[37]
CHT/chitosan/PSS/PEM		81.67		96.11	
Chitosan/PES	Zn^2+^	NA	50	80.00	[38]
	Pb^2+^	NA		90.00	
Chitosan/PES+ polypropylene	Cr^6+^	9.3	75	75.00	[36]
poly(AA-co-VP)@APTES@MCN/PES	Cd^2+^	322.5	NA	78.00	[39]
PEES/n-ZnO/PEG	Cu^2+^	44.3	103	93.27	[40]
	Zn^2+^	50.6		89.15	
	Ni^2+^	57.3		85.81	
	Cd^2+^	60.9		83.19	
GNP/KMnO_4_/PSF	Cr^6+^	NA	103	93.07	[41]
	Fe^3+^	NA		98.75	
Amino- Zr-MOFs-CUF	Pb^2+^	1100	30	61.40	[42]
Ni-Zn MOF/PSF	Pb^2+^	8.85	80	98.00	[43]
BCl + MEUF	As^3+^	NA	0.15	92.80	[44]
DPCl + MEUF				84.10	
Chitosan + MEUF	Cr^6+^	30.73	3	100.00	[45]
Chitosan-SDS + MEUF		53.89	3	98.33	
ABSNa50 + TP220 + MEUF	Cu^2+^	NA	100	100.00	[46]

**Table 2:** Removal of heavy metals by NF membranes.

NF Membrane Systems	Pressure (MPa)	Zeta Potential (mV)	Flux (L/m^2^ h)	Heavy Metal	Concentration of Metal (mg/L)	Rejection (%)	Ref.
PEG/α-CD/ PAN membranes	NS	NS	5.9	Ru^3+^	50	83.10	[52]
			5.5	Ag^+^		88.00	
			5.9	Co^2+^		90.20	
TFN-PEI-GO	0.5	NS	70.3	Pb^2+^	500	91.00	[53]
				Cu^2+^		96.00	
				Cd^2+^		96.00	
TFN/UiO-66-NH_2_	0.8	NS	60.41	Pb^2+^	500	99.00	[54]
CysCNC TFN	1 ± 0.1	−23.5 ± 1.2	16	Cu^2+^	200	89.20	[49]
				Pb^2+^		87.00	
PEI-Cu(II)-CSP	1	NS	8.1	Ni^2+^	5	83.15	[55]
				Cd^2+^		75.50	
PEI-Cu(II)-CSP	0.8	NS	18.1	Cd^2+^ Zn^2+^ Ni^2+^ Pb^2+^	5	86.00 87.20 89.50 89.90	[56]
MPD/TMC/DWCNT + PES	0.5	NS	109	Na^+^	000	96.48	[57]
PDA/Zr-MOF-TFN FO membranes	NS	−28	13.2 14 13.8	Ni^2+^ Pb^2+^ Cd^2+^	2000	99.00 99.00 96.00	[58]
NH_2_-MIL-101 (Al)/0.35 wt% ZnO +Cs-UF matrix	0.5	+11	1.5 1.6	Mg^2+^ Ca^2+^	1000	90.10 80.49	[59]
PEI/PAA hollow fiber membranes	NA	−27.6	29.6 ^1^	Cu^2+^	100	94.20	[60]
TA/Fe^3+^PEI hollow fiber membranes	0.1	+37	23.18	Fe^3+^ Cu^2+^ Mn^2+^ Zn^2+^	4000	95.70 94.10 92.90 91.10	[61]
CNFs-co-Cs PES membranes	NS	NS	13.58	Cu^2+^ Cr^6+^ Pb^2+^	>20	97.86 98.40 96.54	[62]

^1^ means permeation flux, others are water flux. NS: no statistics in references; NA: not available in references.

**Table 3:** Comparison of membrane processes.

Membranes	Advantages	Disadvantages	Ref.
PEUF	Recovery and reuse of collected retentate and polymer binding, low operational cost, and high separation efficacy	Requirement of proper pre-treatment process and microligand, and not suitable for industrial wastewater	[4]
MEUF	High separation efficacy, low energy input, and rapid reaction	Generation of secondary pollutants and post-treatment, be costly and unsustainable	[4,32]
Adsorptive MMMs	Low cost, low-pressure requirement, high thermal and chemical strength, and high water yield	Agglomeration and low affinity between the adsorbent materials	[47,116]
NF	High heavy metals rejection at intermediate operating pressure range	Fouling issue, high energy consumption and low water permeability	[4,117]
ENFMs	Easy handling, high selectivity, ease of manufacturing, environmentally friendly and low cost	Limited reusability, fouling effect, hazardous solvents, and highly time-consuming unstable morphology	[77,80,118]
MOFs membranes	Tunable nature of the pore, selectivity, appended functionality, fast capture kinetics and regeneration capacity	Particle agglomeration, difficult to separate and recover, high cost	[83,117]
IIMs	High adsorption capacity, high extraction efficiency, easy preparation and low cost, reusability and high species formation	Weak recognition ability, desorption difficulties, and poor reusability	[89]
Mxenes	High-level hydrophilicity, chemical stability, tunable chemistry and negative surface charges	High cost, stability issue	[99,103]

## Data Availability

Not applicable.
